# Stimulation of Adenosine A_2B_ Receptor Inhibits Endothelin-1-Induced Cardiac Fibroblast Proliferation and α-Smooth Muscle Actin Synthesis Through the cAMP/Epac/PI3K/Akt-Signaling Pathway

**DOI:** 10.3389/fphar.2017.00428

**Published:** 2017-06-30

**Authors:** Sarawuth Phosri, Ajaree Arieyawong, Kwanchai Bunrukchai, Warisara Parichatikanond, Akiyuki Nishimura, Motohiro Nishida, Supachoke Mangmool

**Affiliations:** ^1^Department of Pharmacology, Faculty of Pharmacy, Mahidol UniversityBangkok, Thailand; ^2^Division of Cardiocirculatory Signaling, Okazaki Institute for Integrative Bioscience (National Institute for Physiological Sciences), National Institutes of Natural SciencesAichi, Japan; ^3^Department of Translational Pharmaceutical Sciences, Graduate School of Pharmaceutical Sciences, Kyushu UniversityFukuoka, Japan; ^4^Precursory Research for Embryonic Science and Technology, Japan Science and Technology AgencyKawaguchi, Japan

**Keywords:** A_2B_ receptor, Akt, cAMP, cardiac fibroblast, Endothelin-1, Epac, PI3K, α-SMA

## Abstract

**Background and Purpose:** Cardiac fibrosis is characterized by an increase in fibroblast proliferation, overproduction of extracellular matrix proteins, and the formation of myofibroblast that express α-smooth muscle actin (α-SMA). Endothelin-1 (ET-1) is involved in the pathogenesis of cardiac fibrosis. Overstimulation of endothelin receptors induced cell proliferation, collagen synthesis, and α-SMA expression in cardiac fibroblasts. Although adenosine was shown to have cardioprotective effects, the molecular mechanisms by which adenosine A_2_ receptor inhibit ET-1-induced fibroblast proliferation and α-SMA expression in cardiac fibroblasts are not clearly identified.

**Experimental Approach:** This study aimed at evaluating the mechanisms of cardioprotective effects of adenosine receptor agonist in rat cardiac fibroblast by measurement of cell proliferation, and mRNA and protein levels of α-SMA.

**Key results:** Stimulation of adenosine subtype 2B (A_2B_) receptor resulted in the inhibition of ET-1-induced fibroblast proliferation, and a reduction of ET-1-induced α-SMA expression that is dependent on cAMP/Epac/PI3K/Akt signaling pathways in cardiac fibroblasts. The data in this study confirm a critical role for Epac signaling on A_2B_ receptor-mediated inhibition of ET-1-induced cardiac fibrosis via PI3K and Akt activation.

**Conclusion and Implications:** This is the first work reporting a novel signaling pathway for the inhibition of ET-1-induced cardiac fibrosis mediated through the A_2B_ receptor. Thus, A_2B_ receptor agonists represent a promising perspective as therapeutic targets for the prevention of cardiac fibrosis.

## Introduction

Cardiac fibrosis is one of the major causes of heart failure and contributes to the abnormality of cardiac functions. After cardiac injury, a number of paracrine and neuronal hormone systems including angiotensin II (Ang II), ET-1, and transforming growth factor beta (TGFβ) promote cardiac fibroblast activation, leading to fibroblast proliferation, ECM overproduction, and myofibroblast differentiation (Porter and Turner, 2009; Kong et al., 2014). Myofibroblast is an active cell which characterized by overproduction of α-SMA (Tomasek et al., 2002). The accumulation of ECM proteins and the differentiation of fibroblast into myofibroblast lead to the replacement of cardiac myocytes with fibrotic scar tissue, resulting in cardiac fibrosis (Brown et al., 2005; Kong et al., 2014). Cardiac fibrosis disrupts the communicational and functional of cells in the heart, making the abnormality of contractility and heart rhythm and also accelerates the cardiac remodeling process which elicits the detrimental effects of the heart and increases the risk of heart diseases (Brown et al., 2005; Porter and Turner, 2009; Kong et al., 2014).

The blood and tissue levels of ET-1 have been found to be increased in patients with heart failure. In addition, overstimulation of ET_A_ receptors induced cardiac fibrosis associated with cardiac dysfunction and heart failure (Mueller et al., 2011). In contrast, development of cardiac fibrosis and hypertrophy in mouse models is impaired in mice with vascular endothelial cell-specific ET-1 deficiency (Adiarto et al., 2012). Hence, ET-1 contributes to the pathogenesis of heart diseases. In cardiac fibroblasts, treatment with ET-1 induced cell proliferation, collagen synthesis, and α-SMA expression as a hallmark of cardiac fibrosis (Nishida et al., 2007). Myocardial fibrosis in turn is important in cardiac remodeling. In this present study, we used ET-1 as a profibrotic agent for a model of cardiac fibrosis studying in the cells. It is crucial to discover the new therapeutic approaches to prevent and reverse underlying cardiac fibrosis induced by ET-1.

Inhibition of fibroblast proliferation, fibroblast differentiation, and collagen synthesis might be the therapeutic target for prevention of cardiac remodeling and fibrosis. The functions of cardiac fibroblasts are regulated by several autocrine/paracrine factors including adenosine. Several studies demonstrated that adenosine could attenuate the adverse impact of cardiac remodeling probably due to the fact that adenosine inhibits several cardiac fibroblast functions For instance, the beneficial effects of adenosine on cardiac fibroblasts consist of decreased collagen synthesis, cell proliferation and differentiation (Dubey et al., 1998; Villarreal et al., 2009). In addition, adenosine decreased collagen deposition in the infarcted area and attenuated an increase in collagen volume and collagen I expression in non-infarcted area in animal model of myocardial infarction (MI) (Villarreal et al., 2003). Furthermore, stimulation of adenosine receptors leads to myocardial protection after ischemia (Baxter et al., 1994) and cardiac remodeling (Villarreal et al., 2003). Adenosine receptors belong to the G protein-coupled receptors (GPCRs) superfamily, denoted A_1_, A_2A_, A_2B_, and A_3_ receptors (Fredholm et al., 2000). All subtypes of adenosine receptors are endogenously expressed in rat cardiac fibroblasts. A_1_ and A_3_ receptors are coupled to Gα_i/o_ to inhibit AC activity, whereas A_2A_ and A_2B_ receptors are coupled to Gα_s_ to activate AC activity (Klinger et al., 2002).

The actions of adenosine on inhibition of collagen synthesis are not mediated through A_1_ and A_3_ receptors signaling but likely through A_2_ receptors. For instance, stimulation of adenosine A_2B_ receptor inhibited PDGF-BB-induced DNA synthesis, cellular proliferation and collagen synthesis in adult rat cardiac fibroblasts (Dubey et al., 2001). In addition, stimulation of A_2_ receptors also inhibited Ang II-induced collagen synthesis in adult rat cardiac fibroblast (Villarreal et al., 2009). Moreover, an *in vivo* study showed that stimulation of A_2B_ receptor attenuated fibrosis and remodeling in rat hearts after MI (Wakeno et al., 2006). Thus, A_2_ receptors may play an important role in regulation of cardiac fibrosis. Although many studies have demonstrated the antifibrotic effects of adenosine, it is still unclear which adenosine receptor subtypes are involved in the inhibition of ET-1-induced cell proliferation and α-SMA expression in cardiac fibroblasts.

After agonist binding, A_2_ receptors couple to Gα_s_ proteins, which results in an activation of AC and subsequently elevates the cAMP levels. The pathways associated with adenosine-induced cAMP elevation include the activation of PKA and Epac pathways (Klinger et al., 2002). In adult rat cardiac fibroblasts, stimulation of A_2_ receptors leads to the elevation of cAMP and that the resultant cAMP inhibits Ang II-induced collagen synthesis via a PKA-independent, Epac-dependent pathway (Villarreal et al., 2009). Moreover, activation of Epac by the A_2B_ receptor induced phosphorylation of ERK1/2 in human umbilical vein endothelial cells (HUVECs) (Fang and Olah, 2007). In addition, stimulation of A_2B_ receptor can stimulate ERK1/2 activity in human mast cell line (MHC-1 cells), suggesting that activation of ERK1/2 pathways are important steps in adenosine A_2B_ receptor-dependent stimulation of IL-8 synthesis (Feoktistov et al., 1999). Furthermore, stimulation of A_2_ receptors inhibited Ang II-induced collagen synthesis via Epac/PI3K signaling pathway in cardiac fibroblasts (Villarreal et al., 2009).

However, the exact molecular mechanisms by which adenosine inhibited ET-1-induced cardiac fibrosis remains unclear. Therefore, we investigated the signaling pathways of A_2_ receptors on the inhibition of ET-1-induced fibroblast proliferation and α-SMA production in neonatal rat cardiac fibroblasts.

## Materials and Methods

### Reagents and Plasmid Construction

7-(2-phenylethyl)-5-amino-2-(2-furyl)-pyrazolo-[4,3-e]-1,2,4-triazolo[1,5-c]pyrimidine (SCH58261), 2-Phenylaminoa denosine (CV1808), ET-1, ESI-09, and LY294002 were purchased from Sigma-Aldrich (St. Louis, MO, United States). Dulbecco’s Modified Eagle Medium (DMEM), fetal bovine serum (FBS), 0.25% trypsin-EDTA solution, and penicillin/streptomycin (P/S) solution were purchased from Gibco (Grand Island, NY, United States). Collagenase A was purchased from Roche Diagnostics (Indianapolis, IN, United States). Akt inhibitor IV, Forskolin, PKA inhibitor 14-22 amide (PKI), DDA, 6-Bnz-cAMP, and 8-[4-[((4-Cyanophenyl)carbamoylmethyl)oxy]phenyl]-1,3-di(n-propyl)xanthine hydrate (MRS1754) were purchased from Calbiochem (San Diego, CA, United States). 8-pCPT-2-O-Me-cAMP-AM (ESCA-AM) was purchased from Tocris Biosciences (Bristol, United Kingdom). SCH58261, Akt inhibitor IV, ESI-09, LY294002, MRS1754, and ESCA-AM were dissolved in dimethyl sulfoxide (DMSO) whereas ET-1, forskolin, PKI dissolved in distilled water when preparing the stock solutions. CV1808 was dissolved in 50% ethanol solution. Aliquots of stock solutions were stored at the correct temperature.

### Neonatal Rat Cardiac Fibroblasts Isolation and Culture

Animal studies were approved by the Committee for Animal Care and Use of the Faculty of Pharmacy, Mahidol University (Protocol No. PYR004/2556 and PYR003/2560) and conducted in accordance with the Guide for the Care and Use of Laboratory Animals published by the United States National Institutes of Health (NIH Publication No. 85-23, revised 1996). Isolation of cardiac fibroblasts was prepared as previously described (Nishida et al., 2007). Briefly, neonatal Sprague-Dawley rats were euthanized by decapitation and the hearts were removed and placed on ice. The atria of the hearts are quickly removed and the remaining hearts were cut into small pieces and digested by collagenase type A (Roche Diagnostics). The digested cells were plated on 10-cm dishes at 37°C for 2–3 h. Cardiac fibroblasts were attached to the bottom of the dishes during incubation time, whereas non-adherent myocytes were removed by changing the cultured media. Cardiac fibroblasts were cultured in DMEM containing 10% FBS, and 1% (v/v) P/S solution (Gibco). The purity of acquired cardiac fibroblast was then confirmed by both cellular morphology (thin and triangular cells) and immunostaining. Cardiac fibroblasts in the first and second passages were used for all experiments. The medium was changed to serum-free DMEM overnight before stimulation.

### Cell Proliferation Assay

Proliferation of cardiac fibroblasts was assessed by MTT (3-(4, 5-Dimethylthiazol-2-yl)-2, 5-Diphenyltetrazolium Bromide) assay, which is based on the transformation of yellow tetrazolium salt of MTT by mitochondria of living cells to an insoluble formazan salt. Cells were plated in 96-well plates (5,000 cells/well) in DMEM containing 1% FBS and 1% P/S solution and incubated for 24 h. The cells were pretreated without or with DDA (10 μM), PKI (10 μM) or ESI-09 (10 μM) for 30 min. Cells were treated with 10 μM CV1808 for 1 h, and then added 20 nM ET-1 for 24 h. After stimulation, cells were added 100 μl of MTT solution (1 mg/ml) to each well and incubated for 3 h at 37°C. Formazan crystals were dissolved with DMSO and plates were read using an Infinite M200 microplate reader (Tecan) at wavelength of 570 nm. The absorption value directly represents relative cell numbers. The percentage of cell viability was calculated according to this equation:

The % of cell viability = [(Absorbance of treated cells)/(Absorbance of control cells)]×100

### Western Blotting

Cells were plated in 6-well plates (2 × 10^5^ cells/well) in DMEM containing 10% FBS and 1% P/S solution. Following stimulation, cells were washed with PBS and solubilized in Triton X-100 lysis buffer (20 mM Tris-HCl, pH 7.4, 0.8% Triton X-100, 150 mM NaCl, 2 mM EDTA, 10% glycerol, 100 μM PMSF, 5 μg/ml aprotinin and 5 μg/ml leupeptin). Protein concentration of cell lysates was determined using a Bio-Rad protein assay kit with bovine serum albumin (BSA) as standard. Samples were mixed with loading buffer and denatured by heating at 95°C before separation by SDS-PAGE. Separated proteins were transferred to PVDF membrane (Bio-Rad) and subjected to immunoblotting with primary antibodies to α-SMA (1:2000; Sigma-Aldrich), phospho-Akt (1:2000; Cell signaling), Akt (1:2000; Cell signaling) and GAPDH (1:2000; Cell signaling). Blotts were visualized with an HRP-conjugated secondary antibodies and a chemiluminescence detection system (GE Healthcare).

### Quantitative Real-Time PCR

The extraction of RNA from cardiac fibroblasts was performed by using GeneJET RNA purification kits (Thermo scientific). Quantitative mRNA analysis was performed by the Mx 3005p Real Time PCR system (Stratagene, La Jolla, CA) using the KAPA SYBR FAST One-step RT-qPCR kits (KAPA biosystems, Wilmington, MA, United States) following the manufacturer’s instructions. Primers sequences are shown in Supplementary Table S1. Relative mRNA expression levels were calculated by the comparative cycle threshold (C_T_) method and normalized to the GAPDH level. Comparison of mRNA expression in each sample was calculated based on the differences in ΔC_T_ of individual samples (ΔΔC_T_).

### Detection of α-SMA by Immunofluorescence Microscopy

Cardiac fibroblasts (1 × 10^4^ cells/well) were plated in 35-mm glass dishes in DMEM containing 10% FBS and 1% P/S solution. After treatment, cells were washed with PBS and then fixed cell with 4% paraformaldehyde and kept at 4°C for overnight. Cell were washed with PBS and then added 0.1% Triton-X in PBS for 5 min. Cell were washed again with PBS and then added 1% BSA in PBS and stored at room temperature for 30 min. After that, cells were added with anti-α-SMA antibody (1:250) and incubated at 37°C for 1 h. Cells were washed twice with PBS, added goat anti-mouse antibody (Alexa Fluor 488), and incubated at 37°C for 1 h. After 1 h, cells were treated with 5 μg/ml DAPI for 10 min and washed twice with PBS. The α-SMA protein in the cells was visualized by fluorescence microscope (Inverted microscope, Olympus IX 81).

### Measurement of cAMP Level by ELISA

Cardiac fibroblasts (2 × 10^5^ cells) were plated in 6 well plates in DMEM containing 10% FBS and 1% P/S solution, and then starved in serum-free DMEM for 2 days. Cells were pretreated with 0.5 mM 3-isobutyl-1-methylxanthine (IBMX) for 1 h, and then stimulated with 10 μM CV1808 for 30 min. After treatment, cells were washed with PBS and then lysed with 0.1 M HCl containing 0.15% Triton X-100. The cAMP level of cell lysate was measured using cyclic AMP ELISA kit (Cayman), following the manufacturer’s instructions.

### Statistical Analysis

Data are presented as mean ± SEM. The statistical analysis was determined using Student’s *t*-test and one-way analysis of variance (ANOVA) followed by Tukey’s test. *P* < 0.05 was considered statistically significant.

## Results

### Stimulation of A_2_ Receptors by CV1808 Inhibits ET-1-Induced Cell Proliferation and α-SMA Expression

**FIGURE 1:**
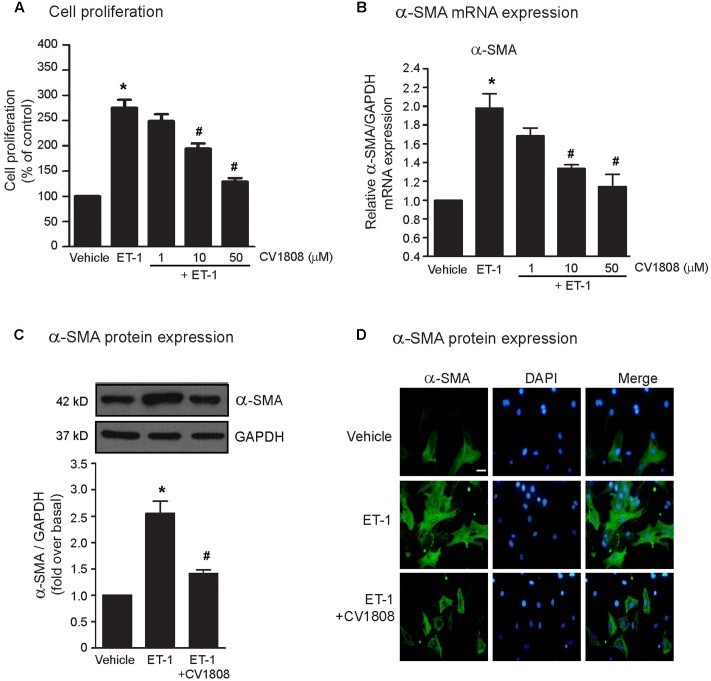
Effects of CV1808 on ET-1-induced cell proliferation and α-SMA synthesis in cardiac fibroblast. **(A,B)** Cardiac fibroblasts were pretreated with various concentrations of CV1808 for 1 h and then stimulated with 20 nM ET-1 for 24 h **(A)** or 12 h **(B)** at 37°C. **(A)** Cell proliferation was quantified by MTT assay. The data were expressed as the percentage relative to the non-treated group, and shown as mean ± SEM (*n* = 4). **(B)** Relative α-SMA mRNA levels were quantified and shown as the mean ± SEM (*n* = 4). ^∗^*P* < 0.05 vs. control; *^#^P* < 0.05 vs. ET-1. **(C,D)** Cardiac fibroblasts were pretreated without or with 10 μM CV1808 for 1 h and then stimulated with 20 nM ET-1 for 24 h at 37°C. **(C)** Relative α-SMA protein levels were quantified and shown as the mean ± SEM (*n* = 4). *^∗^P* < 0.05 vs. vehicle; *^#^P* < 0.05 vs. ET-1. **(D)** Cells were incubated with anti-α-SMA antibody followed by goat anti-mouse antibody (Alexa Fluor 488). The α-SMA was visualized by fluorescent microscope. Cells were stained for α-SMA (green) and nuclear staining of nucleus with DAPI (blue). Bar, 10 μm.

We first assessed the effects of CV1808 (2-Phenylaminoadenosine; a selective adenosine A_2_ receptor agonist) on inhibition of ET-1-induced cell proliferation. Cardiac fibroblasts were pretreated with various concentrations of CV1808 (1–50 μM) before treatment with ET-1 (20 nM) for 24 h. Stimulation of endothelin (ET) receptors with ET-1 resulted in a significant increase in the number of cells compared to that of control (vehicle) group (**Figure 1A**). Pretreatment with CV1808 inhibited ET-1-induced cell proliferation in a dose-dependent manner that shown the maximal effect at the concentration of 50 μM (**Figure 1A**), demonstrating that stimulation of A_2_ receptors inhibits ET-1-induced cardiac fibroblast proliferation. The α-SMA expression is the hallmark of myofibroblast differentiation. We next determined the effects of CV1808 on ET-1-induced mRNA and protein expressions of α-SMA in cardiac fibroblast. We found that treatment with ET-1 significantly increased α-SMA mRNA expression compared with the expression observed in the control (vehicle) group (**Figure 1B**). Moreover, ET-1 treatment caused a substantially increase in α-SMA protein expression, detected by either western blotting (**Figure 1C**) or fluorescent microscopy (**Figure 1D**). Interestingly, pretreatment with CV1808 significantly decreased ET-1-induced α-SMA mRNA and protein levels (**Figures 1B,C**). These data indicate that an antifibrotic effect of CV1808 is one of the potential mechanisms for treatment of cardiac fibrosis under sustained ET receptor stimulation.

### cAMP Is Required for Antifibrotic Effects of CV1808

**FIGURE 2:**
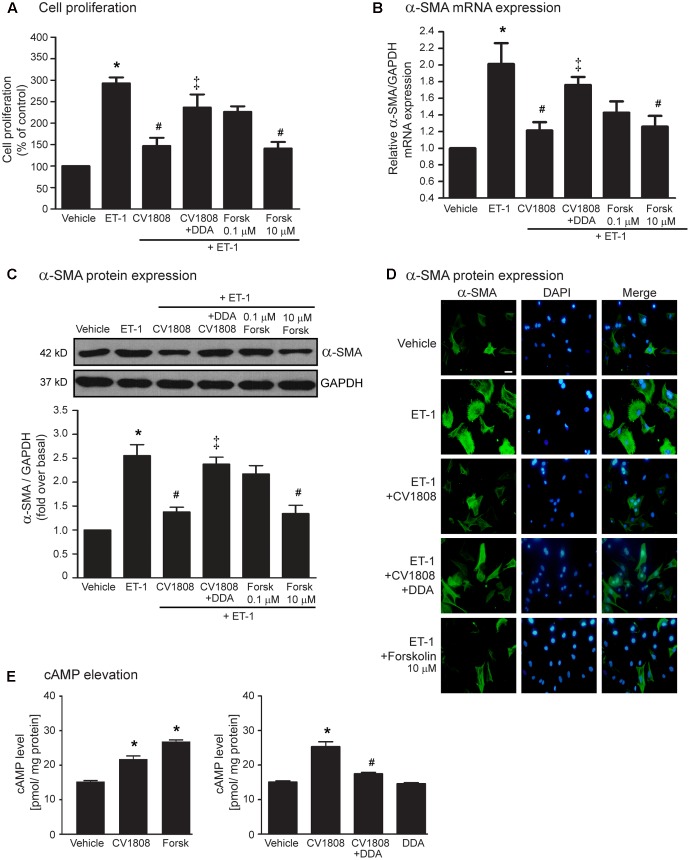
CV1808 mediated-inhibition of ET-1-induced cell proliferation and α-SMA synthesis is cAMP dependent. **(A–D)** Cardiac fibroblasts were pretreated without or with 10 μM DDA (AC inhibitor) for 1 h. After 1 h, cells were treated with vehicle (control), 10 μM CV1808, or forskolin (Forsk; AC activator) for 1 h and further stimulated with 20 nM ET-1 for 12 h **(B)** or 24 h **(A,C,D)** at 37°C. **(A)** Cell proliferation was quantified by MTT assay. The data were expressed as the percentage relative to the non-treated group, and shown as mean ± SEM (*n* = 4). *^∗^P* < 0.05 vs. vehicle; *^#^P* < 0.05 vs. ET-1; ^‡^*P* < 0.05 vs. CV1808+ET-1. **(B,C)** Relative α-SMA mRNA **(B)** and protein **(C)** levels were quantified and shown as the mean ± SEM (*n* = 4). *^∗^P* < 0.05 vs. vehicle; *^#^P* < 0.05 vs. ET-1; ^‡^*P* < 0.05 vs. CV1808+ET-1. **(D)** Cells were incubated with anti-α-SMA antibody followed by goat anti-mouse antibody (Alexa Fluor 488). The α-SMA was visualized by fluorescent microscope. Cells were stained for α-SMA (green) and nuclear staining of nucleus with DAPI (blue). Bar, 10 μm. **(E)** (Left) Cells were treated with 10 μM CV1808, forskolin, or vehicle for 30 min. (Right) Cells were pretreated without or with 10 μM DDA for 1 h. After 1 h, cells were treated with vehicle (DMSO), or 10 μM CV1808 for 30 min. The cAMP levels were measured using cyclic AMP ELISA kit and shown as [pmol/ mg protein] (*n* = 4). *^∗^P* < 0.05 vs. vehicle; *^#^P* < 0.05 vs. CV1808.

Stimulation of A_2_ receptors by CV1808, which couples with Gα_s_ protein, leads to the elevation of cAMP levels via activation of AC activity. Because A_2_ receptor agonists (e.g., NECA, and adenosine) were also shown to increase cAMP levels in the heart and cAMP is a regulator of fibroblast functions (Insel et al., 2012), we investigated whether the inhibition of ET-1-induced cardiac fibrosis by CV1808 is dependent on cAMP. We demonstrated that stimulation of A_2_ receptors with CV1808 significantly increased the cAMP levels, which had effects similar to those of forskolin (an AC activator) (**Figure 2E**). Pretreatment with DDA (an AC inhibitor) completely blocked CV1808-mediated cAMP elevation in cardiac fibroblasts (**Figure 2E**). The inhibitory effects of CV1808 on ET-1-induced cell proliferation and the expression of α-SMA was blocked by DDA (**Figures 2A–D**). In addition, treatment with forskolin at 10 μM was able to inhibit ET-1-induced cell proliferation and the expression of α-SMA as shown by the similar results to those of CV1808 (**Figures 2A–D**). Thus, stimulation of A_2_ receptors delivers potent antifibrotic effects through cAMP signaling by decreasing the cell proliferation and inhibiting α-SMA production induced by ET-1.

### Stimulation of A_2_ Receptors Inhibits ET-1-Induced Cardiac Fibrosis in a PKA-Independent, Epac-Dependent Manner

**FIGURE 3:**
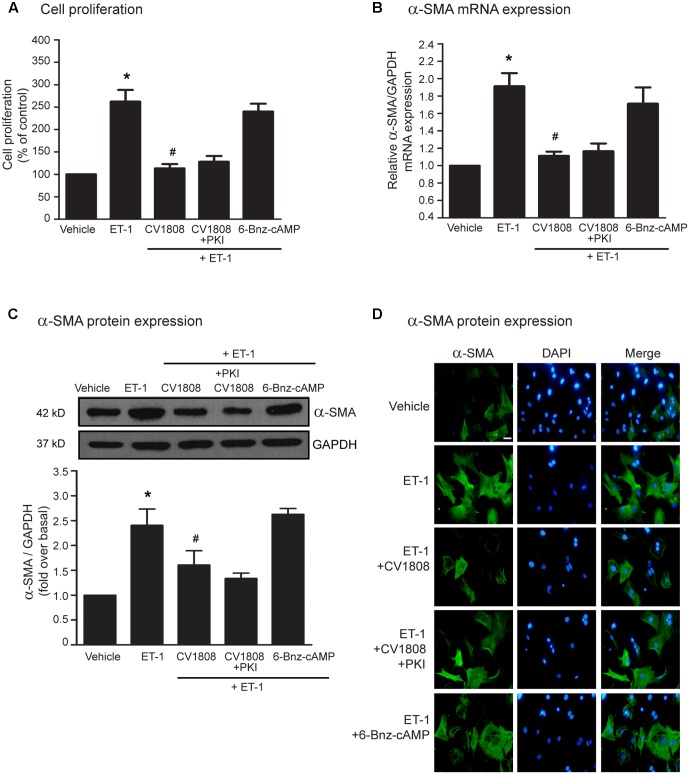
CV1808 mediated-inhibition of ET-1-induced cell proliferation and α-SMA synthesis is independent of PKA. **(A–D)** Cardiac fibroblasts were pretreated without or with 10 μM PKI (PKA inhibitor) for 1 h. After 1 h, cells were treated with vehicle (control), 1 μM 6-Benz-cAMP (PKA activator), or 10 μM CV1808 for 1 h and further stimulated with 20 nM ET-1 for 12 h **(B)** or 24 h **(A,C,D)** at 37°C. **(A)** Cell proliferation was quantified by MTT assay. The data were expressed as the percentage relative to the non-treated group, and shown as mean ± SEM (*n* = 4). *^∗^P* < 0.05 vs. vehicle; *^#^P* < 0.05 vs. ET-1. **(B,C)** Relative α-SMA mRNA **(B)** and protein **(C)** levels were quantified and shown as the mean ± SEM (*n* = 4). *^∗^P* < 0.05 vs. vehicle; *^#^P* < 0.05 vs. ET-1. **(D)** Cells were incubated with anti-α-SMA antibody followed by goat anti-mouse antibody (Alexa Fluor 488). The α-SMA was visualized by fluorescent microscope. Cells were stained for α-SMA (green) and nuclear staining of nucleus with DAPI (blue). Bar, 10 μm.

To examine the antifibrotic effects following A_2_ receptor stimulation, we measured the cell proliferation and the mRNA and protein expression levels of α-SMA after treatment with CV1808. At least 2 downstream effectors are activated by cAMP, PKA and Epac. As we shown that cAMP is necessary for antifibrotic effects of CV1808, we next investigated whether the PKA-dependent pathway, the Epac-dependent pathway, or both, plays a role in A_2_ receptor-mediated antifibrotic actions in cardiac fibroblast. As shown in **Figure 3A**, treatment with PKI (a specific PKA inhibitor) did not block the effects of CV1808 on inhibition of ET-1-induced cell proliferation. In addition, treatment with 6-Bnz-cAMP (a selective PKA activator) had no effect on ET-1-induced cell proliferation (**Figure 3A**). Moreover, blockade of PKA activity by PKI did not alter the inhibitory effects of CV1808 on the mRNA and protein expressions of α-SMA (**Figures 3B–D**). In contrast, treatment with 6-Bnz-cAMP alone did not inhibit ET-1-induced α-SMA mRNA and protein expressions in cardiac fibroblasts. Taken together, these data indicated A_2_ receptor-mediated antifibrotic effect is independent of PKA.

**FIGURE 4:**
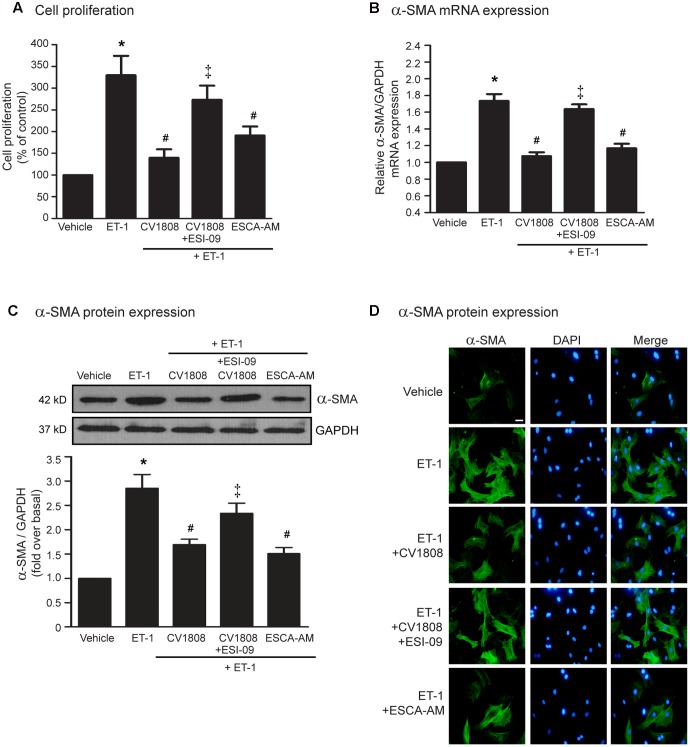
Stimulation of A_2_ receptors inhibits ET-1-induced cell proliferation and α-SMA synthesis in an Epac-dependent pathway. **(A–D)** Cardiac fibroblasts were pretreated without or with 10 μM ESI-09 (Epac inhibitor) for 1 h. After 1 h, cells were treated with vehicle (control), 10 μM ESCA-AM (Epac activator), or 10 μM CV1808 for 1 h and further stimulated with 20 nM ET-1 for 12 h **(B)** or 24 h **(A,C,D)** at 37°C. **(A)** Cell proliferation was quantified by MTT assay. The data were expressed as the percentage relative to the non-treated group, and shown as mean ± SEM (*n* = 4). *^∗^P* < 0.05 vs. vehicle; *^#^P* < 0.05 vs. ET-1; ^‡^*P* < 0.05 vs. CV1808+ET-1. **(B,C)** Relative α-SMA mRNA **(B)** and protein **(C)** levels were quantified and shown as the mean ± SEM (*n* = 4). *^∗^P* < 0.05 vs. vehicle; *^#^P* < 0.05 vs. ET-1; ^‡^*P* < 0.05 vs. CV1808+ET-1. **(D)** Cells were incubated with anti-α-SMA antibody followed by goat anti-mouse antibody (Alexa Fluor 488). The α-SMA was visualized by fluorescent microscope. Cells were stained for α-SMA (green) and nuclear staining of nucleus with DAPI (blue). Bar, 10 μm.

Epac acts as an essential effector of cAMP signaling. We next used either ESCA-AM (a specific Epac activator) or ESI-09 (a specific Epac inhibitor) to examine whether Epac is necessary for A_2_ receptor-mediated antifibrotic effect. We found that blockade of Epac activity by ESI-09 significantly abolished the inhibitory effects of CV1808 on ET-1-induced cell proliferation and α-SMA mRNA and protein expressions (**Figures 4A–D**). Moreover, activation of Epac by 8-pCPT-2-O-Me-cAMP (Epac selective cAMP analog; ESCA-AM) significantly inhibited ET-1-induced cell proliferation and α-SMA production which had effects similar to those of CV1808 (**Figures 4A–D**). Collectively these data suggested that Epac is required for A_2_ receptor-mediated inhibition of cardiac fibrosis induced by ET-1.

### Stimulation of A_2_ Receptors Exhibits Antifibrotic Effects through PI3K-Akt Signaling Pathway

**FIGURE 5:**
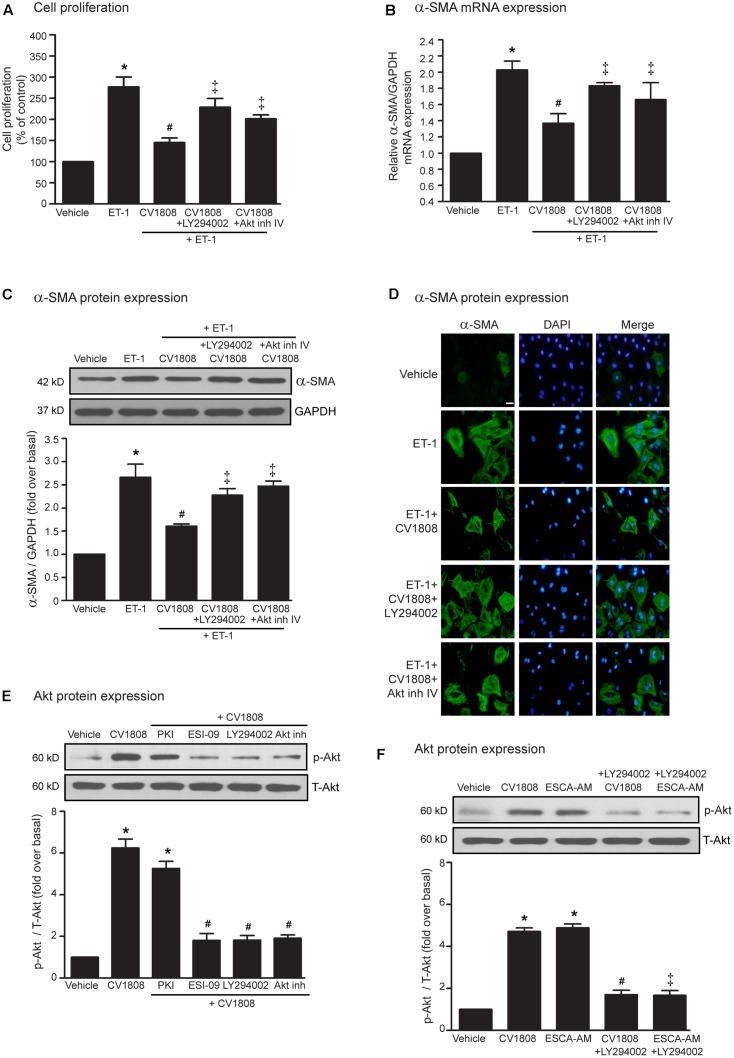
Stimulation of A_2_ receptors exhibits antifibrotic effects through PI3K/Akt-dependent pathway. **(A–D)** Cardiac fibroblasts were pretreated without or with either 10 μM LY294002 (PI3K inhibitor) or 1 μM Akt inh (Akt inhibitor IV) for 1 h. After 1 h, cells were treated with vehicle (control), or 10 μM CV1808 for 1 h and further stimulated with 20 nM ET-1 for 12 h **(B)** or 24 h **(A,C,D)** at 37°C. **(A)** Cell proliferation was quantified by MTT assay. The data were expressed as the percentage relative to the non-treated group, and shown as mean ± SEM (*n* = 4). *^∗^P* < 0.05 vs. vehicle; *^#^ P* < 0.05 vs. ET-1; ^‡^*P* < 0.05 vs. ET-1+CV1808. **(B,C)** Relative α-SMA mRNA **(B)** and protein **(C)** levels were quantified and shown as the mean ± SEM (*n* = 4). *^∗^P* < 0.05 vs. vehicle; *^#^ P* < 0.05 vs. ET-1; ^‡^*P* < 0.05 vs. ET-1+CV1808. **(D)** Cells were incubated with anti-α-SMA antibody followed by goat anti-mouse antibody (Alexa Fluor 488). The α-SMA was visualized by fluorescent microscope. Cells were stained for α-SMA (green) and nuclear staining of nucleus with DAPI (blue). Bar, 10 μm. **(E)** Cells were pretreated without or with 10 μM PKI, 10 μM ESI-09, 10 μM LY294002 or 10 μM Akt inhibitor for 1 h before treatment with vehicle (control), or 10 μM CV1808 for 30 min at 37°C. **(F)** Cells were pretreated with 10 μM LY294002 for 1 h before treatment with either 10 μM ESCA-AM, or 10 μM CV1808 for 30 min at 37°C. **(E,F)** Cell lysates were immunoblotted with anti-phospho-Akt and anti-Akt antibodies. The Akt activation was quantified, expressed as fold increase over vehicle, and shown as the mean ± SEM (*n* = 4). *^∗^P* < 0.05 vs. vehicle; *^#^P* < 0.05 vs. CV1808; ^‡^*P* < 0.05 vs. ESCA-AM.

Previous studies have been reported that treatment with NECA (adenosine receptor agonist) can induce the phosphorylation of Akt (Villarreal et al., 2009) and Akt serves as an important downstream effector of PI3K and Epac; hence, we next further investigate whether PI3K and Akt plays a role on A_2_ receptor-mediated inhibition of ET-1-induced cell proliferation and α-SMA expression. We found that inhibition of PI3K by using LY294002 and inhibition of Akt by using Akt inhibitor IV were able to block the inhibitory effects of CV1808 on ET-1-induced cell proliferation (**Figure 5A**) and α-SMA mRNA and protein synthesis (**Figures 5B–D**).

As we known that Akt is the downstream effector of Epac signaling, we next investigate whether blockade of PKA, Epac, PI3K activities are able to inhibit CV1808-induced Akt phosphorylation. After stimulation of A_2_ receptors with CV1808, the levels of phosphorylated Akt (p-Akt) markedly increased in cardiac fibroblasts, whereas CV1808-mediated Akt phosphorylation was significantly inhibited by either ESI-09 (Epac inhibitor) or LY294002 (PI3K inhibitor) (**Figure 5E**). In contrast, blockade of PKA activity by PKI had no effect on CV1808-mediated Akt phosphorylation.

In addition, treatment with ESCA-AM (Epac activator) resulted in a significant increase in p-Akt level as similar with CV1808 (**Figure 5F**). In contrast, both CV1808 and ESCA-AM were unable to increase the Akt phosphorylation when blockade of PI3K activity using LY94002, suggesting that Akt activation by A_2_ receptor agonist reflects an Epac-dependent that occurs through PI3K activity. Taken together, these results suggested that both PI3K and Akt are involved in the Epac-dependent A_2_ receptors signaling pathway.

### Stimulation of the A_2B_ Receptor Subtype Is Responsible for Inhibition of ET-1-Induced Cell Proliferation and α-SMA Synthesis

**FIGURE 6:**
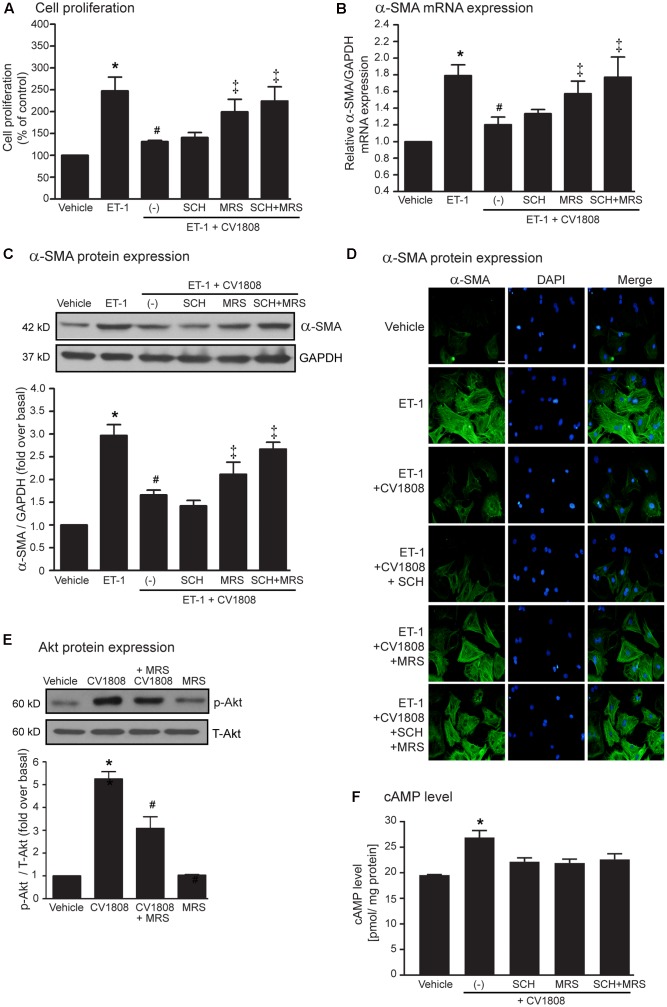
Stimulation of the A_2B_ receptor inhibits ET-1-induced cell proliferation and α-SMA synthesis. **(A–D)** Cardiac fibroblasts were pretreated without or with either 0.5 μM SCH58261 (SCH; A_2A_ receptor antagonist), 0.5 μM MRS1754 (MRS; A_2B_ receptor antagonist), or both for 1 h before treatment with vehicle (control), or 10 μM CV1808 for 1 h and further stimulated with 20 nM ET-1 for 12 h **(B)** or 24 h **(A,C,D)** at 37°C. **(A)** Cell proliferation was quantified by MTT assay. The data were expressed as the percentage relative to the non-treated group, and shown as mean ± SEM (*n* = 4). *^∗^P* < 0.05 vs. vehicle; *^#^P* < 0.05 vs. ET-1; ^‡^*P* < 0.05 vs. ET-1+CV1808. **(B,C)** Relative α-SMA mRNA **(B)** and protein **(C)** levels were quantified and shown as the mean ± SEM (*n* = 4). *^∗^P* < 0.05 vs. vehicle; *^#^P* < 0.05 vs. ET-1; ^‡^*P* < 0.05 vs. ET-1+CV1808. **(D)** Cells were incubated with anti-α-SMA antibody followed by goat anti-mouse antibody (Alexa Fluor 488). The α-SMA was visualized by fluorescent microscope. Cells were stained for α-SMA (green) and nuclear staining of nucleus with DAPI (blue). Bar, 10 μm. **(E)** Cells were pretreated without or with 0.5 μM MRS1754 for 1 h. After 1 h, cells were treated with vehicle (DMSO), or 10 μM CV1808 for 30 min. Cell lysates were immunoblotted with anti-phospho-Akt and anti-Akt antibodies. The Akt activation was quantified, expressed as fold increase over vehicle, and shown as the mean ± SEM (*n* = 3). *^∗^P* < 0.05 vs. vehicle; *^#^P* < 0.05 vs. CV1808. **(F)** Cardiac fibroblasts were pretreated with 1 μM SCH, 1 μM MRS, or SCH plus MRS for 1 h before stimulation with 10 μM CV1808 for 30 min. The cAMP levels were measured using cyclic AMP ELISA kit and shown as [pmol/ mg protein] (*n* = 4). *^∗^P* < 0.05 vs. vehicle.

We next used a selective A_2A_ receptor antagonist (SCH58261), and a selective A_2B_ receptor antagonist (MRS1754) to determine which A_2_ receptor subtypes are associated in the inhibition of ET-1-induced cell proliferation, and α-SMA synthesis. Both SCH58261 and MRS1754 are able to inhibit A_2_ receptors-mediated cAMP elevation, suggesting that these antagonists effectively blunt the receptor signaling (**Figure 6F**). We found that MRS1754 significantly antagonized the inhibitory effect of CV1808 on ET-1-induced cell proliferation, whereas SCH58261 had no effect (**Figure 6A**). In addition, MRS1754, but not SCH58261, also reduced the inhibitory effect of CV1808 on ET-1-induced α-SMA mRNA and protein expressions (**Figures 6B–D**). Furthermore, treatment with MRS1754 significantly inhibited CV1808-mediated Akt phosphorylation, confirming that stimulation of A_2B_ receptor plays an important role on Akt activation (**Figure 6E**). Taken together, these data demonstrated that stimulation of A_2B_ receptor inhibited ET-1-induced cardiac proliferation and α-SMA expression through the Akt signaling pathway.

## Discussion

In this present study, our findings provide an essential role for cAMP/Epac/PI3K/Akt signaling on A_2_ receptor-mediated antifibrotic effects in cardiac fibroblasts. Stimulation of A_2_ receptors inhibits ET-1-induced cell proliferation and myofibroblast differentiation by suppressing α-SMA expression. PI3K and Akt, the downstream effectors of Epac signaling, are necessary for these effects. Moreover, A_2_ receptor-mediated antifibrotic action appears to be predominantly mediated by the A_2B_ receptor subtype.

Cardiac fibroblasts are not only involved in the normal myocardial functions, but also in the cardiac remodeling that responds to cardiac injury and pathological conditions, including MI and heart failure (Kong et al., 2014). In pathophysiology of cardiac fibrosis, cardiac fibroblasts play the important role in multiple processes, which comprising of cardiac fibroblast proliferation, migration, myofibroblast differentiation, and deposition of ECM proteins (Baudino et al., 2006). Differentiating fibroblasts (or myofibroblasts) are highly active cells that overexpress of α-SMA, and increase proliferation and secretory properties (Tomasek et al., 2002). Inhibition of cell proliferation and myofibroblast differentiation might represent the potential therapeutic targets for prevention of cardiac remodeling and fibrosis.

ET-1 plays a central role in heart diseases and is involved in the pathogenesis of cardiac fibrosis. ET-1 is upregulated in animal model of cardiac fibrosis (Yamamoto et al., 2000). ET-1 can stimulate cell proliferation, ECM deposition and production, and expression of markers of myofibroblast phenotype such as α-SMA (Kuruvilla et al., 2007; Nishida et al., 2007). In addition, treatment with ET receptor antagonist reduced cardiac remodeling in models of infarctive and hypertensive cardiac fibrosis (Mulder et al., 1997; Ammarguellat et al., 2001). Thus, in this study, we utilized ET-1 treatment of cardiac fibroblasts as the means to stimulate proliferation and α-SMA synthesis to examine the molecular mechanisms by which A_2_ receptor stimulation may counteract ET-1-induced cardiac fibrosis.

The growth of cardiac fibroblasts is controlled by several paracrine factors, including adenosine, where it exhibits cardioprotective effects. The biological actions of adenosine and adenosine receptor agonists are receptor-dependent manners (Fredholm et al., 2000). Stimulation of A_1_ and A_3_ receptors inhibits AC activity through activation of Gα_i_ proteins (pertussis toxin-sensitive G protein) and subsequently activates PLC activity, which hydrolyses phosphatidylinositol 4,5 bisphosphate (PIP_2_) into inositol triphosphate (IP_3_) and diacylglycerol. Stimulation of A_2A_ and A_2B_ receptors increases AC activity through Gα_s_ proteins, resulting in the elevation of cAMP and activates its downstream effectors, including PKA and Epac (Schulte and Fredholm, 2003; Epperson et al., 2009; Insel et al., 2012). Previous studies suggested that the antifibrotic effects of adenosine are mediated via A_2_ receptors. For instance, pertussis toxin did not alter the inhibitory effects of adenosine agonist (NECA; 5′-N-ethylcarboxamidoadenosine) on Ang II-induced collagen synthesis in adult rat cardiac fibroblast (Villarreal et al., 2009). Data derived from using of several adenosine receptor agonists and antagonists demonstrated that the inhibitory effects of adenosine agonists (2-chloroadenosine, 5′-N-methylcarboxamidoadenosine) on PDGF-BB–induced DNA synthesis, cell proliferation, and collagen synthesis were significantly blocked by selective A_2_ receptor antagonist, but not selective A_1_ receptor antagonist in adult rat cardiac fibroblasts (Dubey et al., 1997, 1998, 2001). Our data also demonstrated that stimulation of A_2_ receptors with CV1808 (selective A_2_ receptor agonist) also inhibited ET-1-induced fibroblast proliferation and α-SMA expression. Thus, A_2_ receptors, but not A_1_ and A_3_ receptors, is considered as an adenosine receptor subtype that elicits antifibrotic effects via inhibition of ET-1-induced fibroblast proliferation and α-SMA expression. It should be noted that the antifibrotic effects of CV1808 has come from *in vitro* experiment using cardiac fibroblasts. This may not completely apply for clinical use due to lacking of *in vivo* and clinical studies. Therefore more research is necessary for investigating antifibrotic effects and underlying mechanisms of CV108 on prevention of ET-1-induced cardiac fibrosis in animal studies.

Furthermore, our present study has demonstrated only the preventive effects of the A_2B_ receptor on ET-1-induced fibroblast proliferation and α-SMA expression. The restorative roles of A_2B_ receptor following ET-1 administration have not been determined. In addition, in this study we used MTT assay for evaluation of fibroblast proliferation. Even though MTT is a routine cell viability assay for cytotoxicity and cell proliferation, MTT may not completely apply for investigation of antiproliferative effect of CV1808. Thus, it will require additional methods to confirm the antiproliferative action of the A_2B_ receptor.

Although A_2_ receptors play an important role on inhibition of cardiac fibrosis in fibroblasts, a few studies have demonstrated that stimulation of A_1_ receptors exhibits antihypertrophic effect in cardiac myocytes. For instance, *N*-cyclopentyl adenosine (CPA; a selective A_1_ receptor agonist) inhibited the hypertrophic response to ET-1, Ang II, or isoproterenol (Liao et al., 2003). In addition, stimulation of A_1_ receptor inhibited cardiac hypertrophy and prevented heart failure in animal model of pressure-overload (Liao et al., 2003). Thus, adenosine has cardioprotective effects mediated by both A_1_ and A_2_ receptors in the heart.

While many studies have identified an antifibrotic effect of A_2B_ receptor in cardiac fibroblast, *in vivo* studies have demonstrated that inhibition of A_2B_ receptor exhibits the beneficial effects in animal models of cardiac remodeling and fibrosis [reviewed by Vecchio et al. (2017)]. For example, administration of GS-6201 (a selective A_2B_ receptor antagonist) attenuated cardiac enlargement and dysfunction in mouse model of MI (Toldo et al., 2012). In addition, blockade of A_2B_ receptor improved ventricular dysfunction and decreased fibrosis in rat myocardial ischaemia-reperfusion model (Zhang et al., 2014). Thus, further investigations on the precise mechanism underlying the profibrotic effects of the A_2B_ receptor in the heart are still needed.

Adenosine A_2A_ and A_2B_ receptors are expressed in the heart (Epperson et al., 2009). Although A_2A_ and A_2B_ receptors can couple with Gα_s_ proteins and stimulate AC activity, considerable differences exist in their actions and signaling pathways. For instance, stimulation of A_2A_ and A_2B_ receptors enhanced endothelial cell proliferation and stimulation of A_2A_ receptor leads to tube formation. Stimulation of A_2B_ receptor induced VEGF, FGF, and IL-8 production in human endothelial cells via Gα_q_ and activation of PLC, while stimulation of A_2A_ receptor upregulated VEGF in macrophages in a PKA-independent manner (Feoktistov et al., 2002; Leibovich et al., 2002). In addition, adenosine attenuates collagen and protein synthesis through the A_2B_ receptor (Dubey et al., 1998; Chen et al., 2004). Moreover, blockade of A_2B_ receptor and extracellular adenosine deamination enhances pro-fibrotic ATP signaling pathway demonstrated by increasing of α-SMA, TGF-β and collagen synthesis (Lu and Insel, 2013). In concordance with these previous studies, our data demonstrated that stimulation of A_2B_ receptor, but not A_2A_ receptor, inhibited ET-1-induced cell proliferation and α-SMA expression in neonatal rat cardiac fibroblasts. Taken together, A_2B_ receptor is considered an adenosine subtype that exhibit antifibrotic effects in the heart. A_2A_ and A_2B_ receptors share 56% sequence identity and the sequence similarity is relatively low (Sherbiny et al., 2009). It remains unknown whether the C-terminal tails of these two receptor influence subtype specificity and further study is required to determine the divergent cellular response and whether any effectors are assembled within receptor compartmentalization.

Upon binding of its agonist to adenosine receptor, A_2_ receptors couple with Gα_s_ proteins, which results in an activation of AC, followed by cAMP elevation (Shaikh and Cronstein, 2016). Stimulation of adenosine receptor with agonist increased cAMP levels in cardiac fibroblasts (Chen et al., 2004) and cAMP-elevating agents might be useful as antifibrotic treatments. For example, AC activators (e.g., forskolin and cAMP analogs) blocked TGF-β- and Ang II- stimulated collagen synthesis and α-SMA expression by cardiac fibroblasts (Swaney et al., 2005). Forskolin and isoproterenol inhibited serum- and TGF-β -stimulated collagen synthesis by cardiac fibroblasts (Liu et al., 2006). In addition, cAMP-elevating agents inhibited fetal calf serum-induced cardiac fibroblast proliferation (Dubey et al., 1997). Moreover, the cAMP-enhancing agents such as PGE_2_ and forskolin reduced epidermal growth factor (EGF)-induced proliferation of human airway muscle cells (Kassel et al., 2008). Interestingly, overexpression of AC type VI (AC6) increased the production of cAMP and enhanced FSK- and PGE2-mediated inhibition of FBS-induced cell proliferation (Liu et al., 2004). Consistent with previous studies, we showed that treatment with CV1808 (A_2_ receptor agonist) leads to an elevation of cAMP levels that showed the similar effect with forskolin (AC activator). Moreover, treatment with forskolin resulted in the inhibition of ET-1-induced cell proliferation and α-SMA synthesis, while blockade of AC abolished the inhibitory effects of CV1808 on the ET-1-induced these effects. Interestingly activation of A_2B_ receptor inhibits ET-1 production and secretion in guinea-pig tracheal epithelial cells through a cAMP dependent pathway (Pelletier et al., 2000). The relationship between the generation of cAMP and the inhibition of ET-1 production and secretion was also reported in other cells such as rat mesangial cells (Sakamoto et al., 1992). Therefore, further investigations are needed to determine the role for A_2B_ receptor on inhibition of ET-1 production and secretion in cardiac fibroblasts.

Data from our study and others provide important evidences that cAMP appears to be an important effector of A_2B_ receptor-mediated antifibrotic effects in the heart. Even though stimulation of A_2B_ receptor increased cAMP level through Gα_s_ proteins, A_2B_ receptor can couple to the Gα_q_ proteins to activate PLC and increase intracellular Ca^2+^ concentration (Feoktistov et al., 1999; Linden et al., 1999). It will be required further study to investigate whether Gα_q_ is required for A_2B_ receptor-mediated inhibition of ET-1-induced cell proliferation and α-SMA synthesis in the heart.

There are at least two pathways mediated through cAMP such as the PKA-dependent and the Epac-dependent pathway. The pathways associated with downstream actions of adenosine-induced elevation of levels include the stimulation of PKA and Epac activities. Treatment with PKA activator (Sp isomer of 8-bromoadenosine 3′, 5′-cyclic monophosphothioate; Sp-8-Br-cAMP) did not inhibit Ang II-induced collagen synthesis (Villarreal et al., 2009). In addition, blockade of PKA activity by H89 or PKI did not alter the inhibitory effects of NECA on Ang II-induced collagen synthesis (Villarreal et al., 2009), suggesting that PKA activation by cAMP did not involve in A_2_ receptors signaling pathway in cardiac fibroblast. Moreover, A_2B_ receptor-mediated ERK1/2 phosphorylation occurred in a cAMP-dependent, PKA-independent pathway in HUVEC (Fang and Olah, 2007). Our data are consistent with previous studies that the inhibitory effects of CV1808 on ET-1-induced cardiac fibrosis were not affected by inhibition of PKA activity. However, these previous studies and our study are not unlike the study by others in which PKA is necessary for the inhibition of cell proliferation and α-SMA in several types of fibroblasts. For example, stimulation of C2C12 myoblasts with growth factors which control cell proliferation and differentiation is disrupted by cAMP through PKA (Pearson et al., 2006). In addition, TGF-β-induced collagen synthesis and myofibroblast formation is mediated through cAMP/PKA signaling pathway in cardiac and pulmonary fibroblasts (Liu et al., 2004; Swaney et al., 2005). Further evaluation of the divergent cellular response induced by PKA and its compartmentalization remained to be defined.

There are two subtypes of Epac, Epac1 and Epac2 and both are found in the heart. Treatment with Ang II and TGF-β significantly inhibited Epac1 expression, but not Epac2 in cardiac fibroblasts (Yokoyama et al., 2008). This previous study also reported that expression of Epac1 reduced in model of MI rats. Inversely, Epac1 overexpression inhibited TGF-β-induced collagen synthesis (Yokoyama et al., 2008). Moreover, Epac1 expression significantly decreased after treatment with TGF-β in cardiac fibroblasts (Olmedo et al., 2013). In HUVEC, overexpression of Epac1 enhanced A_2B_ receptor-mediated ERK1/2 activation whereas inhibition of Epac1 expression with siRNA reduced this effect (Fang and Olah, 2007), indicating that A_2B_ receptor-mediated Epac activation in HUVEC results in ERK1/2 activation. Moreover, adenosine activates the A_2B_ receptor and the resultant cAMP reduced collagen synthesis through the PKA-independent, Epac-dependent pathway (Villarreal et al., 2009). In lung fibroblasts, treatment with agents that increase cAMP levels inhibited collagen synthesis and cell proliferation that were mediated by Epac (Liu et al., 2004; Racke et al., 2008). Therefore, Epac1 is an important effector of cAMP signaling pathway that elicits the antifibrotic effects in the heart. Moreover, stimulation of A_2B_ receptor can stimulate ERK1/2 activity in human mast cell line (MHC-1 cells), suggesting that activation of ERK1/2 is essential steps in the A_2B_ receptor-mediated IL-8 production in HMC-1 (Feoktistov et al., 1999). The data from previous studies and our study indicated that A_2B_ receptor stimulation can activate cAMP/Epac signaling pathway, leading to ERK1/2 activation in fibroblast cells. Interestingly, our present study demonstrated the new signaling pathway that stimulation of A_2B_ receptor activates cAMP/Epac/PI3K/Akt signaling pathway for inhibition of cell proliferation and α-SMA synthesis induced by ET-1. Our data are in concordance with previous study showing stimulation of A_2_ receptors leads to inhibit Ang II-induced collagen synthesis through Epac/PI3K/Akt signaling pathway in cardiac fibroblast (Villarreal et al., 2009). Moreover, both PKA and Epac play an important role in regulation of neuronal functions, including cell differentiation, proliferation and survival (Nijholt et al., 2008). In cortical neurons, activation of PKA leads to an inhibition of Akt phosphorylation. In contrast, Epac activation increases Akt phosphorylation that is mediated through Rap activation, indicating cAMP/Epac/Akt signaling pathway in the brain (Nijholt et al., 2008). Furthermore, stimulation of Epac activity leads to the PI3K-dependent Akt activation, while PKA stimulation suppresses Akt activity in HEK-293 cells (Mei et al., 2002). Our study also demonstrated cAMP/Epac/PI3K/Akt signaling pathway for A_2B_ receptor-mediated antifibrotic effects by attenuating ET-1-induced fibroblast proliferation and α-SMA expression in cardiac fibroblast. From previous study and our present study, it is likely that Akt is an important downstream effector of Epac in several tissues.

Interestingly, despite A_2B_ receptor mediates Epac/PI3K/Akt signaling pathway in cardiac fibroblast, stimulation of A_3_ receptors also activates PI3K-dependent Akt phosphorylation, leading to the reduction of ERK1/2 phosphorylation, which in turn suppresses cell proliferation in human melanoma cells (A375 cells) (Merighi et al., 2005). Although, it is possible that both PI3K and Akt play a role on antiproliferation after stimulation of different receptor subtypes, further study will be required to determine the precise mechanistic of this pathway and its compartmentation after adenosine receptor stimulation.

**FIGURE 7:**
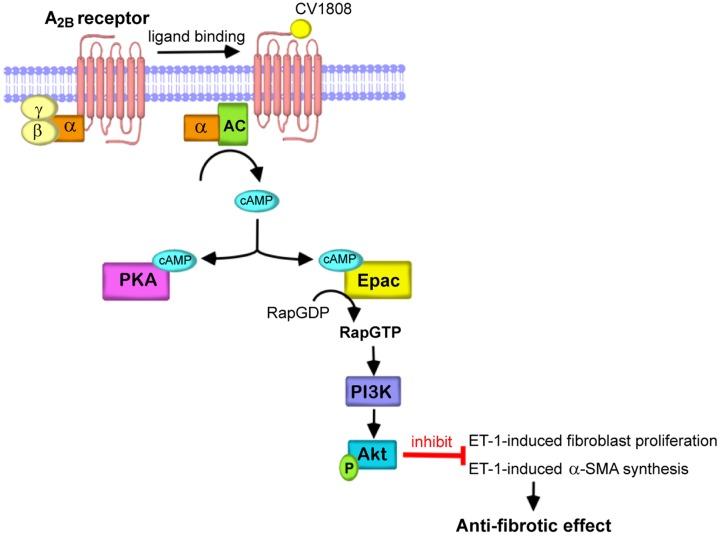
Schematic diagram representing the essential role of Epac/PI3K/Akt for A_2B_ receptor-mediated antifibrotic effects. In cardiac fibroblast, agonist binding to the A_2B_ receptor leads to G protein coupling and activation of AC through Gα_s_ proteins increases the cAMP levels. cAMP directly binds to and activates Epac and subsequently stimulates Epac/PI3K/Akt signaling, which in turn inhibits cell proliferation and α-SMA production induced by ET-1. Therefore, stimulation of A_2B_ receptor elicits the antifibrotic effects in the heart.

Based on the findings of this study, we have identified a new signaling pathway for an inhibition of ET-1-induced cell proliferation and α-SMA expression in cardiac fibroblasts mediated through the A_2B_ receptor (**Figure 7**). Agonist binding to A_2B_ receptor leads to cAMP elevation through Gα_s_ activation. cAMP binds to and activates Epac activity, leading to activation of PI3K and Akt, thereby elicits the antifibrotic effects.

## Author Contributions

SP performed the experiments and data analysis, participated in the study planning and wrote the manuscript; AA performed the experiments, data analysis, and participated in the study planning; KB performed the experiments and data analysis; WP performed the experiments and data analysis; AN performed the experiments and data analysis; MN contributed to the discussion and reviewed/edited the manuscript; and SM carried out the experiments and data analysis, participated in the study planning and wrote the manuscript.

## Conflict of Interest Statement

The authors declare that the research was conducted in the absence of any commercial or financial relationships that could be construed as a potential conflict of interest. The reviewer APF-S and handling Editor declared their shared affiliation, and the handling Editor states that the process nevertheless met the standards of a fair and objective review.

## Abbreviations

AC: adenylate cyclase
6-Bnz-cAMP: N^6^-Benzoyladenosine-3′-5′-cyclic monophosphate
cAMP: cyclic adenosine monophosphate
DDA: 2′, 5′-dideoxyadenosine
ECM: extracellular matrix
Epac: exchange protein directly activated by cAMP
ESCA-AM: Epac-selective cAMP analog acetoxymethyl ester
ET-1: endothelin-1
PI3K: phosphatidylinositol 3-kinase
PKA: protein kinase A
PKI: myristoylated protein kinase A inhibitor amide 14-22
α-SMA: α-smooth muscle actin.
