# Supplementary material for: Stimulation of Adenosine A2B Receptor Inhibits Endothelin-1-Induced Cardiac Fibroblast Proliferation and α-Smooth Muscle Actin Synthesis Through the cAMP/Epac/PI3K/Akt-Signaling Pathway
Source: Front Pharmacol. 2017 Jun 30;8:428. doi: 10.3389/fphar.2017.00428 (PMC5492828; doi:10.3389/fphar.2017.00428)
Supplement: Supplementary file 1 [file Table_1.docx]

**Supporting information**

**Supplemental table 1. Gene specific primers for RT-qPCR (rat)**

| **Gene specific primer** | | **Sequences** |
| --- | --- | --- |
| Rat α-SMA | Sense  Antisense | 5′**-**AGCCAGTCGCCATCAGGAAC-3′  5′-CCGGAGCCATTGTCACACAC-3′ |
| Rat GAPDH | Sense  Antisense | 5′-CAGTCAAGGCTGAGAATG-3′  5′-CGACATACTCAGCACCAGC-3′ |
